# Gene Environment Interactions and Predictors of Colorectal Cancer in Family-Based, Multi-Ethnic Groups

**DOI:** 10.3390/jpm8010010

**Published:** 2018-02-16

**Authors:** S. Pamela K. Shiao, James Grayson, Chong Ho Yu, Brandi Wasek, Teodoro Bottiglieri

**Affiliations:** 1 College of Nursing and Medical College of Georgia, Augusta University, Augusta, GA 30912, USA; 2 College of Business, Augusta University, Augusta, GA 30912, USA; jgrayson@augusta.edu; 3 University of Phoenix, Pasadena, CA 91101, USA; chonghoyu@gmail.com; 4 Center of Metabolomics, Institute of Metabolic Disease, Baylor Scott & White Research Institute, Dallas, TX 75226, USA; brandi.wasekpatterson@bswhealth.org (B.W.-P.); Teodoro.Bottiglieri@BSWHealth.org (T.B.)

**Keywords:** gene–environment interaction, colorectal cancer, predictor, multi-ethnic groups

## Abstract

For the personalization of polygenic/omics-based health care, the purpose of this study was to examine the gene–environment interactions and predictors of colorectal cancer (CRC) by including five key genes in the one-carbon metabolism pathways. In this proof-of-concept study, we included a total of 54 families and 108 participants, 54 CRC cases and 54 matched family friends representing four major racial ethnic groups in southern California (White, Asian, Hispanics, and Black). We used three phases of data analytics, including exploratory, family-based analyses adjusting for the dependence within the family for sharing genetic heritage, the ensemble method, and generalized regression models for predictive modeling with a machine learning validation procedure to validate the results for enhanced prediction and reproducibility. The results revealed that despite the family members sharing genetic heritage, the CRC group had greater combined gene polymorphism rates than the family controls (*p* < 0.05), on *MTHFR* C677T, *MTR* A2756G, *MTRR* A66G, and *DHFR* 19 bp except *MTHFR* A1298C. Four racial groups presented different polymorphism rates for four genes (all *p* < 0.05) except *MTHFR* A1298C. Following the ensemble method, the most influential factors were identified, and the best predictive models were generated by using the generalized regression models, with Akaike’s information criterion and leave-one-out cross validation methods. Body mass index (BMI) and gender were consistent predictors of CRC for both models when individual genes versus total polymorphism counts were used, and alcohol use was interactive with BMI status. Body mass index status was also interactive with both gender and *MTHFR* C677T gene polymorphism, and the exposure to environmental pollutants was an additional predictor. These results point to the important roles of environmental and modifiable factors in relation to gene–environment interactions in the prevention of CRC.

## 1. Introduction

Colorectal cancer (CRC) is a cancer that is preventable by modifying environmental and lifestyle interventions for human ecological development [1,2,3,4,5,6]. Well-defined environmental interventions may improve cancer treatment effects, prevent cancer progression and increase survival through epigenetic mechanisms with gene environment interactions [1,4,5]. Approximately 70% of CRC is related to environmental and lifestyle factors, while about 30% of CRC risk is inheritable with 5% being highly aggressive in cancer progression for metastatic penetrance [7,8,9]. Hence, the most common risks for CRC are preventable by cultivating healthy lifestyles and environments to help keep the human epigenetic environment free from cancers.

For the prevention of various chronic health conditions, the most published genes related to the risk for various racial–ethnic groups is the methylenetetrahydrofolate reductase (*MTHFR*) gene, identified through the genome application framework [10,11,12,13,14]. As the MTHFR enzyme is encoded by the *MTHFR* gene for homocysteine remethylation to methionine, mutations in the *MTHFR* gene are associated with MTHFR enzyme deficiency in humans [15]. Studies have emerged to document the effects of low folate levels and increased CRC risk [12,13,14]. The mechanism of low folate levels and CRC as well as a plethora of major cardiovascular and neurodevelopmental diseases have been associated with the toxic effect of hyperhomocysteinmia [10,12,13,14]. These toxic effects are mediated through one-carbon metabolism enzyme pathways, which are critical to basic biological processes including deoxyribonucleic acid (DNA) and protein methylation, and DNA replication and mutations. *MTHFR* gene is listed as a prototype gene for the application of prevention studies for CRC by the experts at the National Human Genome Research Institute (NHGRI) [16,17]. We examined two loci of *MTHFR* gene polymorphisms, C677T (rs1801133) and A1298C (rs1801131), both are associated with MTHFR enzymatic deficiency resulting in increased homocysteine concentrations [18,19]. The best-characterized *MTHFR* gene polymorphism is a common missense/loss of function mutation of 677C→T, resulting in a thermolabile enzyme variant that has a reduced catalytic activity of 35% for 677 CT and 70% for 677 TT variants, and of nucleotide 1298A→C, resulting in 15% decreased MTHFR activity for 1298 AC and 30% for 1298 CC variants [20,21]. We also investigated three additional genes in the folate-methylation pathways: dihydrofolate reductase (*DHFR*) 19 base pair (*19bp*) (rs70991108) which converts folic acid into methylenetetrahydrofolate (MTHF) as usable folate form [22,23], methionine synthase (*MTR* A2756G, rs1805087) in the methylation cycle, and methionine synthase reductase (*MTRR* A66G, rs1801394) which converts/recycles homocysteine back to usable MTR for the methylation cycle [24,25,26,27]. Together, these five genes play critical roles in the methylation pathways for biological processes in sustaining human health, and polymorphism mutations of these genes would lead to missense and lost functions for the methylation process. Sufficient nutrients related to these genes include folate (vitamin B9) and vitamin B12, as methyl donors, play an integral role in the phenotypic expression of *MTHFR* and related gene mutations in the methylation pathways [18,19,20,21].

Healthy lifestyles and living environments have a major effect on the development of CRC, inducing gene expression changes in the key epigenetics regulatory pathways and affect metabolic processes in colon mucosa [7,10,24]. Lifestyle may play a mediating role with ages in the lifespan for the development of CRC, based on studies that involved the examination of family members developing hereditary CRC [7,8,9]. Thus, studies of gene–environment interactions in families are significant in providing potential insights for developing prevention strategies affecting cancer prevention. Additionally, recent studies including meta-predictions that examined gene–environment interactions consistently presented that increased air pollution is associated with increased gene polymorphisms across various disease conditions, especially for *MTHFR* C677T polymorphisms and genes in the methylation pathways [28,29,30,31,32,33,34,35]. Therefore, the purpose of this study was to examine the key gene–environmental factors affecting the risk associations with CRC, and the interactions among these factors affecting the risks of CRC. In this study, we used three phases of data analytics, including data visualization and identification, data reduction, and model building to validate the predictive models. These analytics included the ensemble method [36,37,38,39], as well as data exploration and generalized regression models for predictive modeling to cross-validate the results [40,41,42,43].

## 2. Results

We used three phases of data analytics, including exploratory family-based analyses adjusting for dependence within the family for sharing genetic heritage [44]. In the first stage of data visualization and understanding, we used bootstrap forest, also known as bagging (i.e. bootstrap aggregating), which is one of the most popular ensemble methods [37,38,39,40]. The ensemble methods are based on the logic of resampling, which is a well-known remedy for small-sample studies. For example, while developing the bootstrapping method in 1983, Diaconis and Efron had only 15 observations [45]. In resampling, the sample is treated as a virtual population and then different subsets are randomly drawn from the sample for multiple analyses. Bias can be observed and corrected by such repeated analyses of random subsets [46]. In the second stage, our strategy was to identify the most influential predictors within three categories of genetic factors, demographic/environmental factors, and lifestyle factors for dimension reduction. We also used generalized regression models for predictive modeling with machine learning validation procedures [47], including significant variables and variables with significant interactions identified through the data visualization of bi-variate interaction profilers, to validate the results for enhanced prediction and reproducibility.

### 2.1. Characteristics of Study Participants

We recruited a total of 54 families, 108 participants, 54 CRC cases and 54 matched family controls. We attempted to match the groups on various demographic factors for this family-based study. The family control group had a younger age because many of the available family members were the offspring of the cancer patients. Table 1 presents the comparisons of key demographic [48], lifestyle health metrics [49,50], and environmental factors [51,52] between the two groups. Parameters that were significantly different between the control and cancer groups included age, gender, and exposure to pollutants (all *p* < 0.05), adjusted for associated blood-related family members [44]. As this was a proof-of-concept study, additional adjustment of *p*-values for multiple testing was not used for the exploratory analyses of related factors.

Other noteworthy factors of importance included sleepiness during day time; cancer patients reported an average of 0.4 more sleepy days than the family controls. Physical inactivity was associated with an elevated risk of cancer (an average of 11 min less active per week in cancer patients than the control group). However, most people were sedentary, only two (3.7%) of the control group and one (1.9%) of the cancer group participants met the recommended 150 minutes or longer physical activity in this study. Additionally, using alcohol was associated with a higher risk of cancer (14.9% more use in the cancer than the control group).

These demographic/lifestyle/environmental factors were compared across the racial–ethnic subgroups (Table 2). The results showed that the Hispanic and the Black samples had higher body mass index (BMI) with greater than 50% of the Hispanic and the Black samples being obese than the White (29.4%) and the Asian (2.4%) samples (*p* < 0.0001). Additionally, there were more Whites than the other three racial groups who drank alcohol (*p* = 0.0001).

Between the two groups, the total gene polymorphism rates of the five chosen genes in the folate methylation pathways ranged from zero to six, with a possible maximum score of 10 if each of the five genes had homozygous polymorphisms. MTHFR enzyme deficiency was calculated by combining the loss of enzyme functions from *MTHFR* C677T (loss of 35% for each of the two T polymorphic alleles) and *MTHFR* A1298C (a loss of 15% for each of the two C polymorphic alleles), a composite score of both *MTHFR* C677T and *MTHFR* A1298C polymorphisms [15]. To decrease the degrees of freedom and increase the power in the statistical testing, the total polymorphism score was recoded into two groups using the median split between <4 and ≥4. Increased polymorphism of the five genes combined was associated with an increased risk of CRC (*p* < 0.05), while no significant difference between the control and cancer groups was noted for each gene alone and the composite score on the MTHFR enzyme deficiency (Table 3). There was a general trend that the cancer group had increased polymorphisms and lesser percentage of wild type alleles for all genes including *MTHFR* C677T, *MTR* A2756G, *MTRR* A66G, and *DHFR* 19bp, except for *MTHFR* A1298C, where the control group had increased polymorphisms and lower wild type alleles compared to the CRC group, which had decreased polymorphisms and higher wild type alleles.

There were significant differences in the presentation of all five gene polymorphisms across the four racial–ethnic groups (all *p* < 0.05, Table 4). For comparison among these racial groups, in general, the Asian and the White samples had more polymorphisms on these five genes combined than the Hispanic and the Black samples. For comparisons among the groups of the individual genes, the Hispanic and the White samples had higher MTHFR enzyme deficiencies (average of 36%) resulting from polymorphisms of *MTHFR* C677T and *MTHFR* A1298C compared to the Asian (27%) and the Black (0%) subgroups. The Asian (88%) and the Black (78%) samples had higher *DHFR* 19 bp deletions than the White (59%) and the Hispanic (48%) samples. The White (79%) and the Black (67%) samples had higher *MTRR* A66G polymorphisms than the Asian (52%) and the Hispanic (26%) samples. Furthermore, the Black (56%) and the White (41%) samples had higher levels of polymorphisms on the *MTR* A2756G gene than the Asian (29%) and the Hispanic (9%) subgroups.

The distribution of the polymorphisms on these five genes for the control and cancer groups and the four racial–ethnic subgroups are further presented in Table 5. We checked the Hardy–Weinberg equilibrium (HWE) analysis of these five genes to assess the distribution equilibrium of the evolutionary mechanisms in population genetics [53], associated with factors such as population migration or stratification and disease association. *MTHFR* A1298C and *DHFR* 19bp had significant (both *p* < 0.05) HWE with disequilibrium, while this was not significant for each of the racial–ethnic subgroups for these two genes. We further checked the distribution of alleles for population-based allele frequencies across the ethnic groups to provide the reference distribution in comparison to our findings (Table 5, http://useast.ensembl.org/index.html; https://www.cdc.gov/genomics/population/genvar/frequencies/mthfr.htm).

### 2.2. Most Influential Predictors per Category—**The Ensemble Method**

Influential predictors were identified in three categories: genetic factors, demographic/environmental factors, and lifestyle factors (as indicated by health metrics) [48,49]. Individual predictors were then selected by using the decision tree methods to build models and then from the rank order of column contributions selecting the most influential variables using the bootstrap forest method [28,29,30,31]. The column contribution is presented using the *G*^2^ statistics, which is derived from the conventional likelihood ratio chi-square statistic, as chi-square is a test of goodness-of-fit between the expected count and the actual account. By the same token, *G*^2^ indicates how well the expected count and actual count classified into that group fit with each other.

The most crucial genetic predictors of cancer (Table 6) appeared to be *MTRR66* polymorphism and MTHFR deficiency. On the rank order of importance among the 10 demographic/environmental factors (Table 7), BMI ranked the highest for importance, followed on the next level by marital status and race, then dropped to the variables of exposure to pollution and gender, then dropped to health insurance coverage and air quality in the community, and finally to variables including the convenience of access health care, air quality in the home, and tobacco smoker in the home. Our exploration found that age alone trumped all other potential predictors. However, this result is not informative because it is a well-known fact that older people are more vulnerable to chronic health issues leading to cancer. This piece of information about age cannot lead to any actionable item because nothing can be done to reverse aging. Thus, age was not included in the exploratory analysis to allow other potential predictors to emerge. Among the 16 lifestyle/health metrics variables (Table 8), after six rows there is a sharp drop of *G*^2^, and therefore stress, physical activity minutes, time using alcohol, spiritual support, sleepiness, and functional role are considered the most important predictors.

In the second stage, dimension reduction, our strategy was to identify the most influential predictors within the three categories of genetic factors, demographic/environmental factors, and lifestyle factors (as indicated by health metrics). To select the most influential predictors within each category, we used the criteria of column contribution and variable importance. Both the ensemble method and the regression methods were run to identify potential predictors in each group and in each category. The misclassification rates of both models were compared to verify the function of a predictive model according to genetic, demographic/environmental factors, and lifestyle categories. For this sample, the random forest models outperformed the original logistic regression analyses for all three domains of factors, as presented by lower misclassification rates (Table 9).

### 2.3. Predictors for Gene–Environment Interaction

The most significant variables for gene–environment interactions were then taken into consideration simultaneously, and Table 10 presents the rank order of important factors by *G*^2^ and a portion of combined bootstrap forest analyses of all three domain factors. *G*^2^ is based on LogWorth and the likelihood ratio chi-square statistics, whereas portion is counted by how often the variable recurs in the repeated analyses. It is important to point out that like using the scree plot in factor analysis, the decision of adopting the most important predictors is based on the overall pattern i.e., how the variable pops out in *G*^2^ relative to others, not an absolute cut-off like the alpha level. It is noteworthy that the first four top predictors are modifiable (BMI, physical activity, sleepiness, and spiritual support). Genetic factors (MTHFR deficiency and *MTRR* A66G polymorphism), which are non-malleable, rank number five and number nine for the total sample.

The role of important predictors in cancer was further examined by racial–ethnic subgroups to explore potential actionable factors per racial–ethnic groups. Table 11 indicates that for Asians (*n* = 42) the number one predictor was sleepiness, then followed by the stress levels, then *MTRR* A66G polymorphism and physical activity levels. The outstanding *G*^2^ suggests that sleepiness and stress trumped all other factors in predicting cancer for Asians. For Hispanics (*n* = 23) the top predictor was spiritual support, which trumped all other factors, as shown in Table 12. For Whites (Table 13, *n* = 34), the most important variables were physical activity, BMI, and alcohol use. Because there were only nine black participants, there was not enough variation for resampling to construct a model using the bootstrap forest method.

### 2.4. Predictive Modeling for Gene–Environment Interactions—Generalized Regression Analysis

Using the most influential variables identified in Section 2.2, two generalized regression models were developed using leave-one-out cross-validation methods to predict the probability of cancer. Generalized regression is also known as penalized regression. As the name implies, the modeling process penalizes complicated models to avoid overfitting. Hence, compared with conventional regression modeling, generalized regression tends to yield an optimal model. In each case, the models were first compared to a logistic regression model with validation for a baseline. For model one the parameter estimates along with the associated *p*-values for the baseline logistic regression results with validation are shown in the left panel of Table 14, including significant interaction terms (BMI interacting with alcohol use) in addition to total gene polymorphism score and other significant parameters. The regularized parameters remaining in the generalized regression elastic net Alkaike’s information criterion (AIC) with correction (AICc) and leave-one-out models are shown in the middle and right panels of Table 14, with the predictor, alcohol use, eliminated from the model as indicated by the zero value for the estimate.

**Figure 1:**
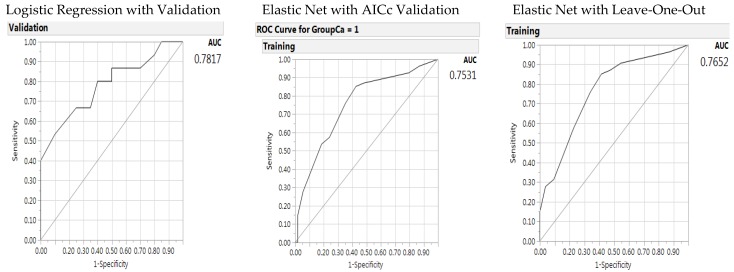
Receiver operating characteristic (ROC) curve and AUC for the baseline logistic regression model (left panel), elastic net with Akaike’s information criteria with correction validation model (middle), and leave-one-out validation model (right panel) on the predictors of colorectal cancer from gene –environment interactions (of total gene polymorphisms).

The predictive performance for the generalized regression elastic net models can be characterized by examining the receiver operating characteristic (ROC) curve and the misclassification rates (Figure 1). The misclassification rate for the baseline logistic regression in the left panel was higher than the other two methods, with a misclassification rate of 0.3714 as compared to 0.2963 and 0.2804. The elastic net validation model outperformed the original logistic regression model on predictive accuracy by lower misclassification rates. The ROC areas under the curve are shown in Figure 1, with the baseline logistic regression model in the left panel with an area under the curve of 0.7817 and the generalized regression elastic net AICc model and leave-one-out model in the middle and right panels with an area under the curve (AUC) of 0.7652. In the elastic net models, alcohol use was the variable to leave out; however, BMI and alcohol had significant interactions. Therefore, as the base of the interactive variable, the BMI variable must remain in the model.

**Figure 2:**
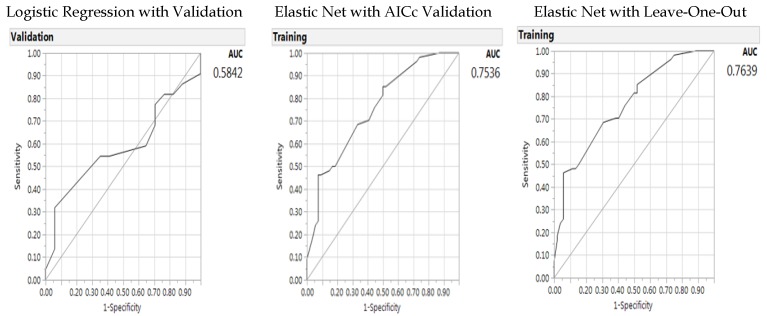
Receiver operating characteristic curve and AUC for baseline logistic regression model (left panel), elastic net with Akaike’s information criteria with correction validation model (middle), and leave-one-out validation model (right panel) on the predictors of colorectal cancer –environment interactions (of single genes).

In a similar way to the previous model, in the second model we used an elastic net AICc validation and with leave-one-out validation with a baseline model of logistic regression with a validation column by including the individual gene parameters and significant interaction terms (gender with BMI, *MTHFR* C677T with BMI. Results of the parameters for the logistic regression are shown in Table 15, and results for the model results are shown in Figure 2 for ROC area under the curve. As before, the generalized regression Elastic Net models outperformed the baseline logistic regression model with better predictive accuracy (lower misclassification rates and larger AUCs). In the elastic net model, BMI was the variable to leave-out; however, BMI and gender status as well as BMI and *MTHFR* C677T polymorphism had significant interactions. Therefore, BMI variable must remain in the model.

In both predictive models of CRC, by either including total gene polymorphisms or individual genes as part of genetic factors of gene–environment interactions, gender (more men than women in the CRC group compared to the control group) and BMI status (more overweight and obese status in the CRC group than the control group) were consistent predictors. In the model where the total gene polymorphism was used for prediction of CRC, alcohol use (more use in the CRC group than the control group) was interactive with BMI status. In the model where the single genes were included for the prediction of CRC, the BMI variable was interactive with both gender and *MTHFR* C677T polymorphism and the exposure to pollution was an additional predictor of CRC in the model when single genes were included. These predictive models were run for each racial–ethnic subgroup. However, we did not observe stable results because of the limited number of samples per racial–ethnic subgroups. Therefore, the subgroup analyses per racial–ethnic subgroups of the predictors of CRC from gene–environment interactions are not presented.

## 3. Discussion

We presented the gene–environment interactions and predictors of CRC by including key genes in the one-carbon metabolism pathways, with environmental and lifestyle factors, by using various analytics to validate the findings across the methods. Using the ensemble method, the most influential factors included gene polymorphisms of *MTRR* A66G and *MTHFR,* and lifestyle factors such as BMI, exposure to pollutants, and gender. Using the most influential factors, the two best predictive models were also generated using the generalized regression models and leave-one-out cross validation methods. With the machine learning approach, these models included a random validation dataset to yield more reliable prediction. For the prediction of CRC, BMI status and gender were consistent predictors in the models. The use of alcohol (more use in the CRC group) interacted with BMI status in predicting CRC. BMI status was also interactive with both gender and *MTHFR* C677T polymorphism in predicting CRC. Also, the exposure to pollutants was an additional predictor of CRC.

While previous studies have presented gene–environment interactions, associating genes in the one carbon metabolism pathways with folate deficiency [24,25,27] and CRC [24,27], new predictive modeling and validation analytics with interactions have become readily available for convenient use through SAS JMP programming (SAS Institute, Cary, NC, USA). Therefore, we included the gene–environment interactions, between the modifiable factors and the genes in our analytic approach, to examine potential epigenetic mechanisms. Overall, the CRC group had increased combined gene polymorphisms than the control group, including *MTHFR* C677T, *MTR* A2756G, *MTRR* A66G, and *DHFR* 19bp, except *MTHFR* A1298C. Additional modifiable factors included BMI status, exposure to pollutants, and alcohol use for CRC risks.

We presented the distributions of the genotype alleles for five genes in the one carbon metabolism pathway for four racial–ethnic groups. In addition to the four gene polymorphisms (*MTHFR* C677T and A1298C, *MTR* A2756G, and *MTR* A66G) that were presented for the CRC cases [24,27], and in numerous meta analyses [10,11,12,13], we included *DHFR* 19 bp deletion as an additional gene in the folate-metabolism pathway. *DHFR* 19 bp in the folate methylation pathway has not been presented for the CRC cases in various ethnic groups before. These four ethnic groups presented different polymorphism patterns for these five genes.

As a proof-of-concept study, to examine gene–environment interactions for cancer prevention, we used the ensemble method, as it is a well-known remedy for small-sample studies to validate the analyses by the random subsets of samples [45]. We further used the generalized regression method integrating significant parameters and bivariate interactions to maximize the model quality with the simplest optimal model. We did not have a sufficient number of subjects for the ethnic subgroups for analyses, especially the Black sample, for most influential predictors or subgroup analyses using the generalized regression model. Therefore, further studies are needed that include larger samples to further validate these findings for various ethnic groups. We presented the very first study cross-validating the findings using both conventional inferential statistics and the ensemble method to predict the risk of CRC. While there are limitations to family-based, case-control designs because of genetic associations among the family members, we used the family-based analysis technique to explore and control for the family associations. Despite these limitations, there are advantages for methodological concerns to include family members in community-based studies. First, the inclusion of family members can enforce the active participation of the family as an ecological unit, and more reliable reporting of modifiable lifestyle or environmental parameters [54,55]. Involving family members in a community-based study can also facilitate support from family members for patients, with a heightened awareness within the family unit of the importance of modifiable lifestyles, thus helping to adopt healthier lifestyles. The validity of research observations is also strengthened in that patient lifestyles are better monitored with the increased awareness of the family unit. Therefore, the rigor and reliability of the data are enhanced, for sustainable interventions with behavioral improvements.

To add to the genetic factors, our results point to a list of modifiable lifestyle and environmental factors [33,34,35,36] in relation to the gene–environment interactions for the prevention of CRC. The top modifiable factors included BMI status, environmental pollution, and alcohol use. Recent studies including metaprediction studies that examined gene–environment interactions consistently presented that increased air pollution is associated with increased gene polymorphism and trends to increased disease risks across various disease conditions, especially for *MTHFR* C677T polymorphisms and genes in the methylation pathways [28,29,30,31,32,33,34,35]. Environmental toxicants such as air pollution and smoking can induce oxidative stress and disregulate reactive oxygen species [28,29,30]. Studies suggested that exposure to oxidative stress caused damage to cellular DNA that leads to mutations, genomic instability, and ultimately malignancy [28,29,30]. From these understandings, future studies may focus on the epigenetics of methyl-donors to detox the hazards from environmental pollution, with healthy lifestyles and weight-based interventions to prevent CRC. Additionally, future research can be designed to examine environmental pollutants and lifestyles with gene–environment interactions in CRC prevention.

## 4. Materials and Method

### 4.1 Study Population and Setting

We included 108 participants, 54 CRC cases and 54 matched family/friend controls by accessing the California Cancer Registry (CCR) database and additional cases through case referrals by the participants. The study was approved by the appropriate Human Subjects Institutional Review Boards (IRB) from the California State Committee for the Protection of Human Subjects for data access through the CCR (CPHS-12-12-1007, approved 2013-2019), and from the local educational institutions (Azusa Pacific University, approved 2013-2015; Augusta University, 806069-7, approved 2015-2018) . To qualify for the study, CRC cases had to be: (1) not at the terminal stage of cancer expecting death within six months, (2) 18–80 years of age, (3) have a family member living with or nearby the case for over one year. Family members must be: (1) 18–80 years of age, (2) not having CRC, (3) not at the terminal stage of other illness expecting death within the six months. Both the case and the family member had to have adequate cognitive and mental capacities, and be willing to participate in the interviews and biological sample for genotyping data collection. The CRC cases were survivors, having been diagnosed with CRC for at least two years by the time the CCR released their data. CRC cases and their families were screened based on the inclusion criteria.

Given that a diverse racial–ethnic population resides in southern California, we targeted to recruit at least five families per racial–ethnic group. representing the proportions of various populations in southern California. Following the approval by the IRBs, CRC cases were screened and randomly selected by systematic stratification based on the racial–ethnic groups from the roster databases provided by the CCR. The qualified cases were contacted through the established procedures as required by the CCR, with an introduction letter followed with phone contact. Moreover, family/friend members residing with or near the CRC cases were recruited along with the CRC cases. Most families were visited at their homes for data collection while a few families visited the campus to participate in data collection.

### 4.2 Demographic/Environmental and Lifestyle Data

Participants were interviewed with items of standardized instruments for health-related lifestyle status [33], following the framework of My Own Health Report (MOHR). The MOHR project included a web-based survey with the list of health metrics including health behaviors and lifestyles. The intent of the MOHR project was to harmonize the national health metrics databases with a minimum dataset in the primary care settings. For this project, the elements of these health metrics included in the MOHR project were included to evaluate the lifestyles in relation to the polygenic one carbon metabolism pathways. Family history, functional capacities, cancer risks and activities, and demographics were collected using the items summarized from the Centers for Disease Control and Prevention (CDC) 1999–2012 National Health and Nutrition Examination Survey and National Health Interview Survey [50]. Community environment and health were collected using the items listed in the integrated prevention framework of Institute of Medicine [51] and World Health Organization [52] for cancer prevention. The family pedigrees were completed with family history data using the standard process established by the Coalition for Health Professional Education in Genetics [48].

### 4.3 Genotyping Data

Data sent to the laboratories were de-identified for subjects. Laboratory staff members were blinded to the case control and other status of the samples to enhance the objectivity of laboratory analyses. The specimens were stored on ice and sent in containers with dry ice via express mail to the laboratory following data collection. Once arrived at the laboratory, specimens were kept frozen in deep freezer at −80 °C freezer until analysis.

Genotyping procedures were described elsewhere earlier [56,57]. Briefly, genomic DNA was isolated from salivary samples using the SK-1 swab and Isohelix collection tubes with dry capsules (Boca Scientific, Boca Raton, FL, USA), and/or from peripheral blood samples using the Qiagen Blood DNA Kit (Qiagen Inc., Valencia, CA, USA). The Taqman technique [56] was used for genotyping of the gene polymorphisms using allele specific fluorescent probes with a StepOnePlus™ real-time polymerase-chain reaction system (Thermo Fisher Scientific, Waltham, MA, USA). Quality control was strictly conducted with four duplicate positive controls and four negative controls loaded in each of 96-well plates. Additionally, genotyping assays were repeated with 10% of the samples that were duplicate with salivary and blood samples, and genotyping results were in 100% agreement for the repeated tests. The results of the genotyping for five genes were shared with the participants within six months or sooner following the data collection, as soon as they became available.

MTHFR enzyme deficiency was calculated by adding up the total loss of enzymatic functions from both *MTHFR* C677T and A1298C polymorphisms, 35% for 677 CT and 70% for 677 TT polymorphisms, and 15% for 1298 AC and 30% for 1298 CC variants [20,21,58]. The total gene mutations from five genes were computed together, with possible ranges of 0–10, with scores of one for heterozygous and two for homozygous polymorphism mutations per each of the five genes included in this study.

### 4.4 Data Analysis

Our data analysis followed three phases of exploratory family-based analysis [44] to adjust for the effects of sharing the genetic heritage within the family, data visualization and understanding, data reduction, and model building using JMP Pro 13 (SAS Institute, Cary, NC, USA) [59,60]. In the first stage of data visualization and understanding, we used bootstrap forest, also known as bagging (i.e., bootstrap aggregating), which is one of the most popular ensemble methods [24,25,26,27]. The ensemble methods are based on the logic of resampling, which is a well-known remedy for small-sample studies [45]. In resampling the sample is treated as the virtual population and then different subsets are randomly drawn from the sample for multiple analyses. Bias can be observed and corrected by such repeated analyses on random subsets [46].

The ensemble method is a resampling technique that synthesizes analyses of many subsets of the original data. This approach is superior to conventional regression modeling because ordinal least square regression or logistic regression analyses tend to yield an overfitted model. Numerous studies have confirmed that the ensemble approach outperforms any single model, such as regression or univariate statistics [61,62,63]. In addition, conventional statistical procedures are limited by the sample size. If the number of parameters to be estimated exceeds the degrees of freedom, the regression model would be highly unstable. The ensemble method is based on machine learning, in which datasets are partitioned and analyzed by different models. Each model is considered a weak learner and the final solution is a synthesis of all these weak learners. When different models are generated by resampling, inevitably some are high bias model (underfit) while some are high variance model (overfit). In the end, the ensemble cancels out these errors. Specifically, each model carries a certain degree of sampling bias, but finally the errors also cancel out each other [62].

In the second stage, dimension reduction, our strategy was to identify the most influential predictors within the three categories of genetic factors, demographic/environmental factors, and lifestyle factors (as indicated by health metrics). To select the most influential predictors within each category, we used the criteria of column contribution and variable importance. Both the ensemble method and the regression methods were run to identify potential predictors in each group in each category. The misclassification rates of both models were compared to verify the function of a predictive model per genetic, demographic/environmental factors, and lifestyle categories. As shown in Table 9, the bootstrap forest model in all three domains outperformed the original logistic regression model with lower misclassification rates per category. Using the bootstrap forest ensemble method, *G*^2^ and the portion of column contribution per variable were used to present the rank order of importance.

In the final stage of model prediction, we used generalized regression to obtain a smaller prediction error [59]. The most significant variables and significant interactions were visualized using the interaction profilers for bi-variate interactions of the three categories of variables, and the final set of significant variables were selected for the tested models. The prediction profiler enables the analyst to ask “what if” questions. Specifically, the analyst manipulates the levels of including different variables to see how the model is changed. By doing so we can understand how the interaction of various factors affect the outcome and the sensitivity of the model. Generalized regression is also known as penalized regression, meaning that the variable selection process penalizes complexity. To get the optimal model, the algorithm imposes a penalty on the model when redundant predictors are included. The index for showing complexity is AIC or AICc [64,65,66], developed by Hirotsugu Akaike [67,68], and is in alignment with Ockham’s razor: All things being equal, the simplest model tends to be the best one; and simplicity is a function of the number of adjustable parameters. Thus, a smaller AIC suggests a more optimal model. Specifically, AIC is a fitness index for trading off the complexity of a model against how well the model fits the data. The general form of AIC is AIC = 2*k* ‒ 2ln*L*, where *k* is the number of parameters and *L* is the likelihood function of the estimated parameters. Increasing the number of free parameters to be estimated improves the model fitness, however, the model might be unnecessarily complex. To reach a balance between fitness and parsimony, AIC not only rewards goodness of fit, but also includes a penalty against over-fitting and complexity. Hence, the most optimal model is the one with the lowest AIC value. Since AIC attempts to find the model that best explains the data with a minimum of free parameters, it is considered an approach favoring simplicity. In this sense, AIC is better than *R*^2^ and adjusted *R*^2^, which always go up as additional variables enter in the model, favoring complexity. However, AIC does not necessarily change by adding variables. Rather, it varies based upon the composition of the predictors and thus it is a better indicator of the model quality [47]. Burnham and Anderson recommend replacing AIC with AICc [64,65], especially when the sample size is small, and the number of parameters is large. Actually, AICc converges to AIC as the sample size gets larger and larger. Hence, AICc should be used regardless of sample size and the number of parameters. The methodology of JMP Pro allows for several classes of modeling estimation methods including lasso, forward selection and elastic net [69], and several validation methods including the ones we chose, AICc validation and leave-one-out cross validation methods, because of their effectiveness for small data sets [70]. Model performance was assessed using misclassification rate (smaller is better), AICc, and the area under the ROC curve.

## Figures and Tables

**Table 1:** Comparison of demographic/environmental factors between family control and cancer groups.

Factors		Control (*n* = 54)	Cancer (*n* = 54)	*p*-Value
Gender	Male Female	14 (25.9%) 40 (74.1%)	25 (46.3%) 29 (53.7%)	0.0275
Marital Status	Married	33 (61.1%)	35 (64.8%)	0.1739
Health Status	Good/Excellent	40 (74.1%)	39 (72.2%)	0.6878
Age	Years (mean ± SD) (range)	47.04 ± 17.16 (18–80)	60.98 ± 10.86 (37– 79)	<0.0001
Body Mass Index	Lean (<20) Normal (20–25) Overweight (25.1–29.9) Obese (>30) mean ± SD (range)	2 (3.7%) 22 (40.7%) 18 (33.3%) 12 (22.2%) 27.8 ± 7.2 (17.2–49.1)	2 (3.7%) 18 (33.3%) 18 (33.3%) 16 (29.6%) 27.4 ± 5.9 (19.0–54.0)	0.8082
Vegetable Intake/Day	≥3 servings	15 (27.8%)	12 (22.2%)	0.6779
Fruit Intake/Day	≥2 servings	27 (50.0%)	24 (44.4%)	0.7345
Whole Grain Intake/Day	≥3 servings	8 (14.8%)	6 (11.1%)	0.7821
Liquid Intake/Day	≥8 cups mean ± SD (range)	16 (29.6%) 5.7 ± 1.6 (4–8)	15 (27.8%) 5.6 ± 1.6 (4–8)	0.9645
Sleepy Days/Week	0 days mean ± SD (range)	10 (19.6%) 2.8 ± 2.4 (0–7)	7 (13.0%) 3.2 ± 2.3 (0–7)	0.7355
Physical Activity	Minutes mean ± SD (range) ≥150 minutes per week	48.1 ± 53.9 (0–360) 2 (3.7%)	37.4 ± 41.8 (0–270) 1 (1.9%)	0.2515
Tobacco Use	Yes	5 (9.3%)	4 (7.4%)	0.7277
Alcohol Use	Yes	24 (44.4%)	32 (59.3%)	0.1478
Stress (0–10)	<5 mean ± SD (range)	32 (59.3%) 4 ± 2.8 (0–10)	31 (57.4%) 4.1 ± 3.0 (0–10)	0.6671
Nervous or Anxious	Not at all	26 (48.1%)	25 (46.3%)	0.9971
Depressed	Not at all	36 (66.7%)	34 (63.0%)	0.3581
Cognitive Capacity	Good/Excellent	46 (85.2%)	45 (83.3%)	0.7418
Functional Capacity	Good/Excellent	49 (90.7%)	45 (83.3%)	0.7027
Role Functions	Good/Excellent	49 (90.7%)	44 (81.5%)	0.4913
Spiritual Support	Good/Excellent	39 (72.2%)	45 (83.3%)	0.2074
Convenience to Healthcare	Good/Excellent	50 (92.6%)	52 (96.3%)	0.2293
Health Insurance Coverage	Good/Excellent	44 (81.5%)	47 (87.0%)	0.1330
Air Quality in Community	Good/Excellent	34 (63.0%)	29 (53.7%)	0.7790
Air Quality at Home	Good/Excellent	12 (22.2%)	13 (24.1%)	0.6859
Tobacco Use by Family Members	Yes	5 (9.3%)	6 (11.1%)	0.7005
Exposure to Pollutants	Yes	5 (9.3%)	14 (25.9%)	0.0202
Race	White Asian Hispanic African	16 (29.6%) 23 (42.6%) 11 (20.4%) 4 (7.4%)	18 (33.3%) 19 (35.2%) 12 (22.2%) 5 (9.3%)	0.8842

The statistically significant values have been highlighted in red. SD: Standard deviation.

**Table 2:** Comparison of demographic, lifestyle, and environmental factors across racial groups.

Factors		White (*n* = 34)	Asian (*n* = 42)	Hispanic (*n* = 23)	African (*n* = 9)	*p*-Value
Gender	Male Female	15 (44.1%) 19 (55.9%)	13 (31.0%) 29 (69.0%)	8 (34.8%) 15 (65.2%)	3 (33.3%) 6 (66.7%)	0.6876
Marital Status	Married	24 (70.6%)	31 (73.8%)	9 (39.1%)	4 (44.4%)	0.0658
Health Status	Good/Excellent	27 (79.4%)	30 (71.4%)	16 (69.6%)	6 (66.7%)	0.3674
Age	Years (mean ± SD) (range)	57.53 ± 2.73 (21–80)	53.55 ± 2.45 (19–79)	49.78 ± 3.31 (18–75)	53.67 ± 5.30 (19–77)	0.3478
Body Mass Index	Lean (< 20) Normal (20–25) Overweight (25.1–29.9) Obese (> 30) mean ± SD (range)	1 (2.9%) 8 (23.5%) 15 (44.1%) 10 (29.4%) 28.7 ± 6.5 (19–50.7)	3 (7.1%) 27 (64.3%) 11 (26.2%) 1 (2.4%) 23.8 ± 2.9 (17.2–30)	0 (0%) 4 (17.4%) 7 (30.4%) 12 (52.2%) 30.8 ± 6.9 (20.6–46)	0 (0%) 1 (11.1%) 3 (33.3%) 5 (55.6%) 32.7 ± 9.3 (24.2–49.1)	<0.0001
Vegetable Intake/Day	≥3 servings	11 (32.4%)	13 (31.0%)	2 (8.7%)	1 (11.1%)	0.1414
Fruit Intake/Day	≥2 servings	15 (44.1%)	22 (52.4%)	10 (43.5%)	4 (44.4%)	0.3406
Whole Grain Intake/Day	≥3 servings	7 (20.6%)	4 (9.5%)	2 (8.7%)	1 (11.1%)	0.3985
Liquid Intake/Day	≥8 cups	10 (29.4%)	12 (28.6%)	7 (30.4%)	2 (22.2%)	0.4805
Sleepy Days/Week	0 days	7 (20.6%)	7 (16.7%)	2 (8.7%)	1 (11.1%)	0.8448
Physical Activity/Week	mean ± SD (range) ≥150 minutes	39.3±35.2 (0–180) 1 (2.9%)	43.9±54.5 (0–360) 1 (2.4%)	54.1±59.2 (0–270) 1 (4.3%)	21.7±17.7 (0–50) 0 (0%)	0.1223
Tobacco Use	Yes	1 (2.9%)	4 (9.5%)	3 (13.0%)	1 (11.1%)	0.5457
Alcohol Use	Yes	27 (79.4%)	13 (31.0%)	14 (33.3%)	2 (22.2%)	0.0001
Stress (0–10)	<5	15 (44.1%)	28 (66.7%)	14 (33.3%)	6 (66.7%)	0.1253
Nervous or Anxious	Not at all	14 (41.2%)	20 (47.6%)	11 (47.8%)	6 (66.7%)	0.4130
Depressed	Not at all	22 (64.7%)	28 (66.7%)	13 (56.5%)	7 (77.8%)	0.8608
Cognitive Capacity	Good/Excellent	31 (91.2%)	34 (81.0%)	19 (82.6%)	7 (77.8%)	0.3889
Functional Capacity	Good/Excellent	27 (79.4%)	40 (95.2%)	19 (82.6%)	8 (88.9%)	0.3398
Role Functions	Good/Excellent	31 (91.2%)	36 (85.7%)	18 (78.3%)	8 (88.9%)	0.4095
Spiritual Support	Good/Excellent	27 (79.4%)	34 (81.0%)	17 (73.9%)	6 (66.7%)	0.5334
Convenience to Healthcare	Good/Excellent	34 (100%)	37 (88.1%)	23 (100%)	8 (88.9%)	0.2321
Health Insurance Coverage	Good/Excellent	32 (94.1%)	30 (71.4%)	22 (95.7%)	7 (77.8%)	0.1175
Air Quality in Community	Good/Excellent	17 (50.0%)	28 (66.7%)	11 (47.8%)	7 (77.8%)	0.4525
Air Quality at Home	Good/Excellent	7 (20.6%)	10 (23.8%)	6 (26.1%)	2 (22.2%)	0.2545
Tobacco Use in Family	Yes	2 (5.9%)	4 (9.5%)	4 (17.4%)	1 (11.1%)	0.5316
Exposure to Pollution	Yes	7 (20.6%)	5 (11.9%)	4 (17.4%)	3 (33.3%)	0.4659

The statistically significant values have been highlighted in red.

**Table 3:** Comparison of gene polymorphisms between family control and cancer groups.

Genes		Control (*n* = 54)	Cancer (*n* = 54)	*p*-Value
*MTHFR* 677	0 (CC) 1 (CT) 2 (TT)	28 (51.9%) 21 (38.9%) 5 (9.3%)	23 (42.6%) 25 (46.3%) 6 (11.1%)	0.6285
*MTHFR* 1298	0 (AA) 1 (AC) 2 (CC)	32 (59.2%) 15 (27.8%) 7 (13.0%)	34 (63.0%) 15 (27.8%) 5 (9.2%)	0.8212
*MTR* 2756	0 (AA) 1 (AG) 2 (GG)	39 (72.2%) 12 (22.2%) 3 (5.6%)	36 (66.7%) 17 (31.5%) 1 (1.8%)	0.3712
*MTRR* 66	0 (AA) 1 (AG) 2 (GG)	28 (52.4%) 18 (33.6%) 7 (13.0%)	19 (35.6%) 25 (46.8%) 10 (18.5%)	0.1842
*DHFR* 19 bp	Del/Del Ins/Del Ins/Ins	20 (37.0%) 17 (31.5%) 17 (31.5%)	13 (24.1%) 25 (46.3%) 16 (29.6%)	0.2188
Total Polymorphism (0–10)	≥4 mean ± SD (range)	16 (29.6%) 3.1 ± 1.3 (0–6)	27 (50.0%) 3.3 ± 1.4 (1–5)	0.0306 0.0819

The statistically significant values have been highlighted in red. Ins: Insertion; Del: Deletion.

**Table 4:** Comparison of gene polymorphisms across racial groups.

Genes		White (*n* = 34)	Asian (*n* = 42)	Hispanic (*n* = 23)	African (*n* = 9)	*p*-Value
*MTHFR* 677	0 (CC) 1 (CT) 2 (TT)	3 (38.2%) 16 (47.1%) 5 (14.7%)	21 (50.0%) 17 (40.5%) 4 (9.5%)	8 (34.8%) 13 (56.5%) 2 (8.7%)	9 (100.0%) 0 (0%) 0 (0%)	0.0362
*MTHFR* 1298	0 (AA) 1 (AC) 2 (CC)	18 (52.9%) 12 (35.3%) 4 (11.8%)	29 (69.1%) 9 (21.4%) 4 (9.5%)	10 (43.5%) 9 (39.1%) 4 (17.4%)	9 (100.0%) 0 (0%) 0 (0%)	0.0885
MTHFR Deficiency	0% 15% 30% 35% 50% 70% mean ± SD (range)	2 (5.9%) 7 (20.6%) 4 (11.8%) 11 (32.4%) 5 (14.7%) 5(14.7%) 35.6 ± 19.5 (0–70)	11 (26.2%) 6 (14.3%) 4 (9.5%) 14 (33.3%) 3 (7.1%) 4 (9.5%) 26.9± 21.4 (0–70)	0 (0.0%) 4 (17.4%) 4 (17.4%) 8 (34.8%) 6 (26.1%) 1 (4.4%) 36.1 ± 13.9 (15–70)	9 (100.0%) 0 (0%) 0 (0%) 0 (0%) 0 (0%) 0 (0%) 0 0	<0.0001
	≥50%	10 (29.4%)	7 (16.7%)	7 (30.4%)	0 (0%)	0.1553
*MTR* 2756	0 (AA) 1 (AG) 2 (GG)	20 (58.8%) 11 (32.4%) 3 (8.8%)	30 (71.4%) 11 (26.2%) 1 (2.4%)	21 (91.3%) 2 (8.7%) 0 (0%)	4 (44.4%) 5 (55.6%) 0 (0%)	0.0475
*MTRR* 66	0 (AA) 1 (AG) 2 (GG)	7 (20.6%) 15 (44.1%) 12 (35.3%)	20 (48.8%) 18 (43.9%) 3 (7.3%)	17 (73.9%) 4 (17.4%) 2 (8.7%)	3 (33.3%) 6 (66.7%) 0 (0%)	0.0002
*DHFR* 19 bp	Del/Del Ins/Del Ins/Ins	3 (8.8%) 17 (50.0%) 14 (41.2%)	24 (57.1%) 13 (31.0%) 5 (11.9%)	4 (17.4%) 7 (30.4%) 12 (52.2%)	2 (22.2%) 5 (55.6%) 2 (22.2%)	<0.0001
Total Polymorphism (0–10)	≥4 mean ± SD (range)	16 (47.1%) 3.62 ± 1.18 (1–6)	21 (50.0%) 3.31 ± 1.37 (0–6)	5 (21.7%) 2.57± 1.24 (1–5)	1 (11.1%) 2.22 ± 0.97 (1–4)	0.0322 0.1244

**Table 5:** Distribution of gene polymorphisms per control and cancer groups across racial groups.

*n* (%)	Control Group					Cancer Group		
Genotypes	0	1	2	*p*-**value** (HWE)	Population Allele Frequency	0	1	2
*MTHFR* 677	CC	CT	TT		% C/T	CC	CT	TT
Total	28 (51.9)	21 (44.4)	5 (9.3)	NS	75/25	23 (42.6)	25 (46.3)	6 (11.1)
White	8 (50.0)	7 (43.8)	1 (6.2)	NS	53/47	5 (27.8)	9 (50)	4 (22.2)
Asian	12 (52.2)	8 (34.8)	3 (13.0)	NS	70/30	9 (47.4)	9 (47.4)	1 (5.2)
Hispanic	4 (36.4)	6 (54.5)	1 (9.1)	NS	55/45	4 (33.3)	7 (58.3)	1 (8.3)
Black	4 (100)	0 (0)	0 (0)	-	91/9	5 (100)	0 (0)	0 (0)
*MTHFR* 1298	AA	AC	CC		% A/C	AA	AC	CC
Total	32 (59.2)	15 (27.8)	7 (13)	0.0314	75/25	34 (63)	15 (27.8)	5 (9.3)
White	7 (43.8)	6 (37.5)	3 (18.8)	NS	85/15	11 (61.1)	6 (33.3)	1 (5.6)
Asian	16 (69.6)	5 (21,7)	2 (8.7)	NS	78/22	13 (68.4)	4 (21.1)	2 (10.5)
Hispanic	5 (45.4)	4 (36.4)	2 (18.2)	NS	84/16	5 (41.7)	5 (41.7)	2 (16.7)
Black	4 (100)	0 (0)	0 (0)	-	85/15	5 (100)	0 (0)	0 (0)
*MTR* 2756	AA	AG	GG		% A/G	AA	AG	GG
Total	39 (72.2)	12 (22.2)	3 (5.6)	NS		36 (66.7)	17 (31.5)	1 (1.8)
White	10 (62.5)	4 (25.0)	2 (12.5)	NS	84/16	10 (55.5)	7 (38.9)	1 (5.5)
Asian	17 (73.9)	5 (21.7)	1 (4.3)	NS	65–91/9–35	13 (68.4)	6 (31.6)	0 (0)
Hispanic	11 (100)	0 (0)	0 (0)	-	19/81	10 (83.3)	2 (16.7)	0 (0)
Black	1 (25)	3 (75)	0 (0)	NS	30–37/63–70	3 (60.0)	2 (40.0)	0 (0)
*MTRR* 66	AA	AG	GG		% A/G	AA	AG	GG
Total	28 (52.6)	18 (33.4)	7 (13)	NS	64/36	19 (35.6)	25 (46.8)	10 (18.5)
White	3 (18.8)	6 (37.5)	7 (43.8)	NS	45/55	4 (22.2)	9 (50.0)	5 (27.8)
Asian	14 (63.6)	8 (36.4)	0 (0)	NS	74/26	6 (31.6)	10 (52.6)	3 (15.8)
Hispanic	10 (90.9)	1 (9.1)	0 (0)	NS	72/28	7 (58.3)	3 (25.0)	2 (16.7)
Black	1 (25.0)	3 (75.0)	0 (0)	NS	73/27	2 (40.0)	3 (60.0)	0 (0)
*DHFR* 19 bp	II	ID	DD		% I/D	II	ID	DD
Total	20 (37)	17 (31.5)	17 (31.5)	0.0068	50/50	13 (24.1)	25 (46.3)	16 (29.6)
White	2 (12.5)	6 (37.5)	8 (50.0)	NS	45–47/53–55	1 (5.6)	11 (61.1)	6 (33.3)
Asian	15 (65.2)	6 (26.1)	2 (8.7)	NS	63/37	9 (47.4)	7 (36.8)	3 (15.8)
Hispanic	2 (18.2)	4 (36.4)	5 (45.4)	NS	58/42	2 (16.7)	3 (25.0)	7 (58.3)
Black	1 (25.0)	1 (25.0)	2 (50.0)	NS	55/45	1 (20.0)	4 (80.0)	0 (0)

HWE: Hardy–Weinberg equilibrium; - not available; NS: Not significant; HWE calculator: http://www.koonec.com/k-blog/2010/06/20/hardy-weinberg-equilibrium-calculator/; http://useast.ensembl.org/index.html; https://www.cdc.gov/genomics/population/genvar/frequencies/mthfr.htm.

**Table 6:** Genetic predictors of cancer.

Term	Number of Splits	*G*^2^	Column Contribution	Portion
*MTRR* A66G Polymorphism	46	1.09506968	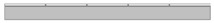	0.3082
MTHFR Deficiency	47	0.82548898	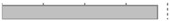	0.2324
*DHFR* 19 bp Deletion	43	0.48910685	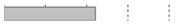	0.1377
*MTR* A2756G Polymorphism	46	0.4855324	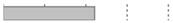	0.1367
*MTHFR* A1298C Polymorphism	42	0.41353505	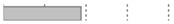	0.1164
*MTHFR* C677T Polymorphism	33	0.24403481	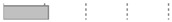	0.0687

**Table 7:** Demographic/environmental predictors of cancer.

Term	Number of Splits	*G*^2^	Column Contribution	Portion
Body mass index	10	6.78930886	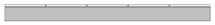	0.3058
Marital Status	7	4.42559099	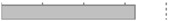	0.1993
Race	7	3.76884353	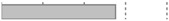	0.1698
Exposure to Pollutants	3	2.12649039	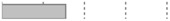	0.0958
Gender	5	1.81587428	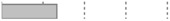	0.0818
Insurance Coverage	3	1.17074973	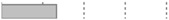	0.0527
Air Quality in the Community	5	1.06350409	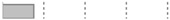	0.0479
Convenience of HealthcareAccess	3	0.52529395	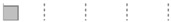	0.0237
Air Quality in the Home	3	0.29975415	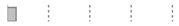	0.0135
Tobacco Smoker in the Home	2	0.21686301	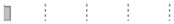	0.0098

**Table 8:** Lifestyle predictors of cancer.

Term	Number of Splits	*G*^2^	Column Contribution	Portion
Stress	27	3.43552989	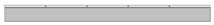	0.1093
Physical Activity	30	3.37660068	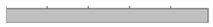	0.1074
Times Using Alcohol	31	3.13235692	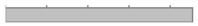	0.0996
Spiritual Support	25	2.91976087	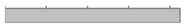	0.0929
Sleepiness	28	2.87042298	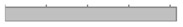	0.0913
Functional Role	22	2.53679611	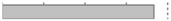	0.0807
Whole Grain Dietary Intake	17	1.92470816	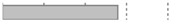	0.0612
Functional Capacity	16	1.81050686	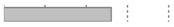	0.0576
Fruits Intake	20	1.52985178	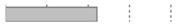	0.0487
Vegetables Intake	20	1.51937688	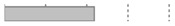	0.0483
Cognitive Capacity	16	1.36910873	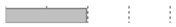	0.0435
Depression	13	1.32859637	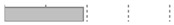	0.0423
Health Status Overall	11	1.25492503	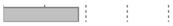	0.0399
Nervous and Anxious	12	1.17885473	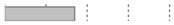	0.0375
Total Liquid Intake	17	0.80560452	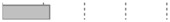	0.0256
Tobacco Smoking	8	0.45134732	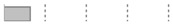	0.0144

**Table 9:** Model comparisons between bootstrap forest and logistic regression.

	Misclassification Rates	
Factors	Bootstrap Forest	Logistic Regression
Demographic–Environmental	0.1942	0.2353
Genetic	0.2019	0.3137
Health Metrics/Lifestyle	0.1584	0.2475

**Table 10:** All predictors of cancer for gene–environment interactions.

Term	Number of Splits	*G*^2^	Column Contribution	Portion
Body mass index	73	2.34801946	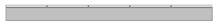	0.1604
Physical Activity	67	1.83265224	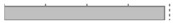	0.1252
Sleepiness	74	1.78325631	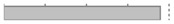	0.1218
Spiritual Support	63	1.75806876	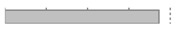	0.1201
MTHFR Deficiency	76	1.66137349	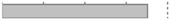	0.1135
Times Using Alcohol	63	1.46035411	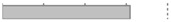	0.0998
Functional Role	65	1.3622703	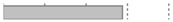	0.0931
Stress	63	1.32282568	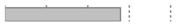	0.0904
*MTRR66* Polymorphism	58	1.10742696	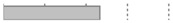	0.0757

**Table 11:** Predictors of cancer for Asians.

Term	Number of Splits	*G*^2^	Column Contribution	Portion
Sleepiness	44	1.44019311	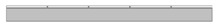	0.2209
Stress	35	1.32619458	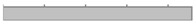	0.2034
*MTRR66* Polymorphism	32	1.02397504	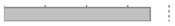	0.1570
Physical Activity, Minutes/Week	38	0.9726631	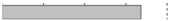	0.1492
Body mass index	25	0.643443	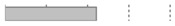	0.0987
MTHFR Deficiency	28	0.46681593	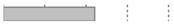	0.0716
Spiritual Support	21	0.29696771	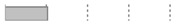	0.0455
Times Using Alcohol	22	0.19012811	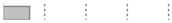	0.0292
Functions in Roles	21	0.16033891	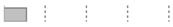	0.0246

**Table 12:** Predictors of cancer for Hispanics.

Term	Number of Splits	*G*^2^	Column Contribution	Portion
Spiritual Support	41	2.51879811	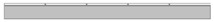	0.4094
Body mass index	18	0.82973589	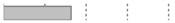	0.1349
Stress	23	0.60238034	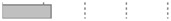	0.0979
Functions in Roles	20	0.54764955	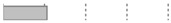	0.0890
Times Using Alcohol	24	0.48150149	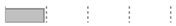	0.0783
Sleepiness	24	0.4798417	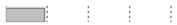	0.0780
Physical Activity, Minutes/Week	18	0.30328675	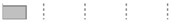	0.0493
MTHFR Deficiency	15	0.22482419	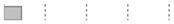	0.0365
*MTRR66* Polymorphism	8	0.16489531	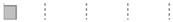	0.0268

**Table 13:** Predictors of cancer for Whites.

Term	Number of Splits	*G*^2^	Column Contribution	Portion
Physical Activity, Minutes/Week	44	1.64218014	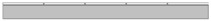	0.1916
BMI	40	1.46162302	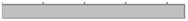	0.1705
Times Using Alcohol	25	1.29832268	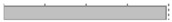	0.1514
Functions in Roles	35	1.15435558	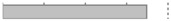	0.1346
MTHFR Deficiency	29	1.11010973	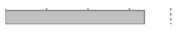	0.1295
Sleepiness	34	0.73467524	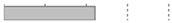	0.0857
Stress	21	0.61669827	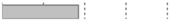	0.0719
*MTRR66* Polymorphism	19	0.32183586	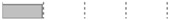	0.0375
Spiritual Support	14	0.23328255	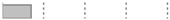	0.0272

**Table 14:** Baseline logistic regression model and generalized regression elastic net models on the predictors of colorectal cancer from gene–environment interactions (of total gene polymorphisms).

	Logistic Regression Original Model with Validation		Generalized Regression Elastic Net Model			
			With AICc Validation		With Leave-One-Out Validation	
Parameters	Estimate	*p* (*X*^2^)	Estimate	*p* (*X*^2^)	Estimate	*p* (*X*^2^)
(Intercept)	−0.2875	0.6144	0.3218	0.4096	0.3486	0.3785
Gender (Male/Female)	1.5023	0.0119	1.2972	0.0074	1.4286	0.0018
BMI * Alcohol Use, Interaction	−2.2790	0.0367	−1.9512	0.0146	−1.2376	0.0062
Total Polymorphisms	−0.7185	0.1865	−1.1444	0.0125	−2.1202	0.0063
BMI	1.3637	0.0602	0.7541	0.1993	0.8991	0.1036
Alcohol Use	0.5468	0.4038	0	1.000	0	1.000
Misclassification Rate	0.3714	-	0.2963	-	0.2804	-
AICc	56.98	-	138.81	-	-	-
AUC	0.7817	-	0.7531	-	0.7652	-

* Denotes Interaction; - not available; AICc: Akaike’s information criterion with corrections; AUC: Area under the curve.

**Table 15:** Baseline logistic regression model and generalized regression elastic net models on the predictors of colorectal cancer from gene –environment interactions (of single genes).

	Logistic Regression Original Model With Validation		Generalized Regression Elastic Net Model			
			Elastic Net Model With AICc Validation		With Leave-One-Out Cross Validation	
Parameters	Estimate	*p*-value (*X*^2^)	Estimate	*p*-value (*X*^2^)	Estimate	*p*-value (X^2^)
(Intercept)	0.5768	0.5445	1.2292	0.0498	1.3171	0.0487
Gender (Male/Female)	3.1964	0.3465	1.4525	0.0049	1.8934	0.0006
Gender (Male/Female) * BMI	−4.2655	0.0039	−1.9736	0.0219	−-2.5539	0.0042
*MTHFR* C677T Polymorphism	−2.3824	0.0345	−0.9065	0.0523	−1.1847	0.0174
*MTHFR* C677T Polymorphism * BMI	2.2401	0.1157	1.2404	0.0667	−1.5750	0.0253
Exposure to Pollution	−0.8194	0.2853	−1.2110	0.0368	−1.2466	0.0458
*MTRR66*	−0.8694	0.1426	−0.6792	0.0975	1.3172	0.0800
BMI	0.8029	0.3465	0	1.000	0	1.000
Misclassification Rate	0.4103	-	0.3241	-	0.3396	-
AICc	85.24	-	140.69	-	-	-
AUC	0.5842	-	0.7536	-	0.7639	-

* Denotes Interaction; - not available; AICc: Akaike’s information criterion with corrections; AUC: Area under the curve.
